# Participatory Design of AI-Based Clinical Decision Support Systems: Scoping Review

**DOI:** 10.2196/88368

**Published:** 2026-10-05

**Authors:** Tabea Rambach, Patricia Gleim, Carolin Heizmann, Philipp Kellmeyer, Christophe Kunze

**Affiliations:** 1 Care & Technology Lab, Furtwangen University, Robert-Gerwig-Platz 1, Furtwangen, Germany, +49 7723920-2976; 2 Data and Web Science Group, School of Business Informatics and Mathematics, University of Mannheim, Mannheim, Germany; 3 Department of Neurosurgery, Human-Technology Interaction Lab, Medical Center and Faculty of Medicine, University of Freiburg, Albertstr. 19, Freiburg im Breisgau, Germany, 49 761-203-97446

**Keywords:** artificial intelligence, AI, participation, participatory design, co-creation, clinical decision support system, CDSS, decision support, challenges, clinical staff, scoping review

## Abstract

**Background:**

AI-based clinical decision support systems (CDSS) can improve diagnostics and treatment decisions, but they are rarely implemented in practice. Barriers include limited integration into clinical workflows, lack of transparency, and insufficient involvement of end users in system design. Participatory and user-centered approaches offer ways to address these challenges by aligning development processes with the needs and routines of clinical staff. However, systematic evidence on how such approaches are applied in the development of AI-based CDSS remains limited.

**Objective:**

Our study examined how participatory approaches are used in the development, piloting, and implementation of AI-based CDSS. We analyzed which user perspectives were included, which participatory methods were applied, how they contributed to technical design, and which ethical, legal, and social implications were addressed.

**Methods:**

This scoping review followed the PRISMA-ScR (Preferred Reporting Items for Systematic Reviews and Meta-Analyses extension for Scoping Reviews) guideline, with a protocol published in advance. A systematic search was conducted in MEDLINE, ACM Digital Library, CINAHL, and PsycInfo for studies published from 2012 onward, complemented by snowballing and manual searches. Primary studies in English or German were included if they involved clinical staff in the development, piloting, implementation, or evaluation of AI-based CDSS. Moreover, 3 independent reviewers conducted screening and data extraction, resolving disagreements by consensus. Data analysis followed JBI methodology and focused on the scope of participation, theoretical and methodological foundations, and reported impacts of participatory approaches.

**Results:**

Of 4318 identified records, 37 met the inclusion criteria. The studies showed broad variation in terminology and methods, most often describing user-centered or iterative processes and less frequently co-design. Physicians were involved in nearly all studies, nurses frequently, and other professional groups only occasionally. Participation mainly supported requirements analysis, adaptation of models to clinical workflows, and the design of explainable interfaces. In several projects, it also influenced data selection, annotation, and visualization. Common barriers included time constraints, limited continuity of participation, and uncertainty toward AI. Ethical, legal, and social aspects were addressed implicitly through themes such as autonomy, responsibility, and traceability, while fairness and bias were rarely discussed.

**Conclusions:**

Participatory processes in AI-based CDSS development should extend across all stages of system design and address not only usability but also data quality, bias, and broader ethical, legal, and social issues. Equal inclusion of nursing and therapeutic expertise is essential to reflect the diversity of clinical decision-making. Clear methodological standards are needed to ensure comparability and to strengthen participation as a genuine co-design process shaping data, models, and values in clinical AI.

## Introduction

### Background

Clinical decision support systems (CDSS) are designed to assist health care professionals in diagnosis, treatment selection, and patient monitoring. These systems aim to improve patient safety and optimize clinical workflows [1-3]. With increasing digitalization and advances in AI, CDSS have increasingly incorporated AI-based technologies to enhance their potential. These systems can access large volumes of digital health data, analyze historical information, continuously learn from new inputs, and provide real-time, personalized recommendations for clinical practice. By identifying patterns in complex datasets, AI enables more precise and individualized decision-making, thereby improving diagnostic accuracy and treatment quality [4,5].

Despite these promises, the implementation of AI-based CDSS in routine clinical care remains limited. Barriers include not only well-known challenges, such as insufficient interoperability, inadequate integration into existing IT infrastructures, and legal uncertainties (eg, data protection and liability), but also a lack of alignment with actual clinical workflows. Many systems fail to reflect the practices, routines, and decision-making logics of their intended users [6,7]. Specific challenges associated with AI-based systems, such as limited data quality, poor model transparency (the “black-box” problem), and ambiguous accountability in AI-supported decisions, further complicate implementation, especially in nursing contexts where documentation is often fragmented and lacks standardization [8,9]. Studies indicate that insufficient user involvement in system development is a key reason for the failure of technological implementations in health care [10].

Participatory approaches offer a promising strategy to address these challenges by involving users early in the development process and systematically integrating experiential knowledge, everyday practice needs, and organizational conditions [11,12]. Such approaches, particularly participatory and user-centered design, aim to develop technologies in close collaboration with future users. Through mutual learning, iterative adaptation, and shared responsibility, these processes aim to produce systems that are not only technically functional but also contextually relevant, acceptable, and usable [13,14]. Participatory and multiperspective methods have proven effective in increasing acceptance and capturing contextual factors in technology design [15-17]. In the field of AI in health care, recent studies show that participatory approaches can enhance data quality, system design, and clinical applicability by incorporating domain knowledge and user expertise [11,12]. Particularly when interpreting complex clinical data, for example, discipline-specific terminology or documentation practices, the user perspective serves as a crucial point of orientation for technical development [11].

At the same time, the literature emphasizes that participatory approaches are not exclusively viewed as beneficial and have also been subject to critical debate. Participation processes may take on a symbolic character (“tokenism”) when participants are formally involved but have limited influence on actual decision-making processes [18]. Moreover, research in design and human-computer interaction (HCI) highlights that user involvement can be instrumentalized, for example, when participant feedback is used to retrospectively legitimize design decisions that have already been made [19]. In addition, participatory processes may be selective, allowing certain stakeholder groups to dominate while other relevant perspectives remain underrepresented [20].

The application of participatory methods in the development of AI-based CDSS remains an emerging and dynamic field of research, presenting specific opportunities and challenges. How can collaboration between AI developers, clinicians, and other health care professionals be structured to accommodate the complexity of algorithmic systems? Which participatory methods are most appropriate for integrating clinical expertise and ensuring alignment with real-world decision-making processes? And how can ethical, legal, and social implications, such as algorithmic transparency, bias mitigation, and accountability, be addressed during system development? These questions are central not only to research but also to the responsible implementation of AI in clinical care. Establishing an iterative feedback loop in which developers and users regularly exchange insights may help ensure that systems evolve in tandem with clinical needs and remain contextually grounded over time.

This review aims to address a significant gap in the existing literature by offering a comprehensive summary of the approaches, methods, and practices of participatory research and development in the context of AI-based CDSS for clinical staff. To achieve this, the review systematically examines empirical studies, their theoretical frameworks, and applied methods for the participatory design of AI-based CDSS. By reviewing the current state of user involvement in the development, design, and implementation of these systems, the review will provide valuable insights into how participatory approaches have been used to date and how they can contribute to successful development and implementation.

### Objectives and Research Questions

The objective of this review is to provide an overview and systematization of participatory approaches from various disciplines for developing, piloting, implementing, and evaluating AI-based CDSS in clinical settings.

The following research questions were addressed:

1. Inclusion of diverse clinical perspectives: Are the different perspectives on underlying clinical problems (eg, by nurses, physicians, and other health care professionals) addressed by any particular CDSS included in the design of the CDSS?
2. Participation as a process: Which participatory approaches are used to develop, pilot, and evaluate AI-based CDSS in health care?
3. Participation in technical aspects of CDSS design: In which ways are participatory methods specifically supporting the development of AI components of CDSS and their performance?
4. Participation for ethical, legal, and social implications: Which ethical, legal, and social implications have been identified in existing projects for participatory development, piloting, implementation, and evaluation processes targeting clinical staff?

## Methods

### Overview

The protocol for this scoping review was previously published in *JMIR Research Protocols* [21]. It outlines the review’s parameters and provides justification and explanations of all methodological steps and decisions. To further ensure rigor, we used the PRISMA-ScR (Preferred Reporting Items for Systematic Reviews and Meta-Analyses extension for Scoping Reviews) checklist [22] and reported it in Checklist 1.

### Search Strategy and Terms

#### Information Sources

To identify relevant papers, we selected databases covering publications in biomedical and health sciences, computer science and information technology, psychology, and nursing and allied health. The following electronic databases were included as information sources: MEDLINE via PubMed, ACM Digital Library, CINAHL, and PsycInfo. These information sources were selected for their broad disciplinary scope, which aligns with the objectives of this review. They provide extensive access to studies on the participatory design and development of AI-based technologies in health care, particularly CDSS.

#### Search Strategy

The databases were searched using combinations of relevant search terms, which were developed and tested for sensitivity before performing the scoping review. We used an iterative approach to develop the search strategy. First, we identified search terms used in previous studies and reviews related to participatory research and AI-based CDSS for clinical staff (particularly relevant are the studies by Kocaballi et al [23], Clar and Wright [24], Kasberg et al [25], Moore et al [26], and Ballard et al [27]). Then, we conducted an initial search in MEDLINE (via PubMed) and CINAHL after analyzing text words (title and abstract) and indexed terms, as suggested by the Joanna Briggs Institute (JBI) methodology for systematic scoping reviews [28,29]. Based on these results, we applied the identified search terms across all databases. Afterward, we checked the references for all included contributions to capture further relevant studies mentioned across the included publications. When necessary, we contacted the corresponding author; this was required only once because the full text was inaccessible.

Multimedia Appendix 1 shows the search strategy for MEDLINE (via PubMed).

To describe the inclusion criteria precisely, we rely on the population, concept, and context scheme [28,29]. The terms were adapted to the basic search particulars (eg, wildcards [*] and truncations) of each electronic database. Textbox 1 shows the most important criteria according to the population, concept, and context scheme.

Textbox 1. **Population, concept, and context criteria used in the scoping review.**

**Population**

- Clinical staff (eg, physicians and nurses).

**Concept**

- Participation, participatory design, cocreation, or co-design.

**Context**

- Development, piloting, implementation, and evaluation of AI-based clinical decision support systems (hybrid and data-driven AI approaches).

**Types of sources**

- Primary research: all study types (eg, qualitative, quantitative, and mixed methods) will be included. Systematic reviews and meta-analyses will be used to conduct manual searches of reference lists to identify additional primary studies.

In line with implementation science literature [30], we define implementation as the integration of AI-based CDSS into routine clinical care beyond prototype development, piloting, or controlled testing.

Reporting on stakeholder involvement in the AI-CDSS field was often sparse and inconsistent in terminology. During the initial screening and full-text assessment, it became apparent that only a limited number of studies explicitly referred to their approach as participatory design or participation, even when clinical stakeholders were involved in development-related activities. To avoid restricting the review to self-labeled participatory studies, we adopted a broader concept of involvement in the concept dimension of the PCC framework. In addition to studies explicitly describing participatory design, co-design, or cocreation, we also included studies reporting consultative forms of user involvement when clinical staff contributed to system development, refinement, piloting, or evaluation. The broader inclusion criteria were already set out in the published review protocol, which also considered related approaches and terms frequently used synonymously with participation or cocreation. It was also based on the staged model of participation by Clar and Wright [24], in which consultation is described as a preliminary stage of participatory influence. At the same time, we are aware that consultative forms of involvement do not necessarily constitute participation in the strict sense, as joint influence or co-determination may remain limited.

At the same time, the review acknowledges that these concepts are not synonymous. Participatory design refers to a design methodology rooted in democratic design traditions and participatory action research emphasizing iterative co-research and co-design with future users to shape technologies, work practices, and design decisions affecting their work [31]. Co-design can be understood as a specific form of cocreation applied to the design process and involving both designers and people who are not trained in design, whereas cocreation refers more broadly to collective creativity shared by 2 or more contributors [13]. In contrast, user-centered design refers to a broad design philosophy and set of methods in which end users inform and influence how a design takes shape, with a strong focus on users’ needs, tasks, and contexts of use, often aiming to improve usability and usefulness [32]. Author-reported terminology was retained descriptively during extraction and not retrospectively harmonized.

To differentiate reported forms of engagement, activities were grouped according to their function within the development process: (1) formative inquiry (eg, interviews, focus groups, and observations), (2) participatory or co-design–oriented activities (eg, co-creation workshops and prototyping), and (3) evaluation-oriented involvement (eg, usability testing, think-aloud procedures, and simulation-based feedback). This analytical distinction was based on the activities reported in the publications. It should not be interpreted as evidence that no earlier or additional involvement had taken place where such processes were not described.

We included studies published from 2012 onward, since this year represents a milestone in the development of deep learning, when the use of deep neural networks in image processing achieved a major breakthrough [33]. The initial database search covered publications published between January 1, 2012, and October 31, 2023. Using the same search strategy, an updated search was conducted for the period from November 1, 2023 to February 18, 2026.

### Eligibility Criteria

The inclusion and exclusion criteria that were applied to the studies are shown in Textbox 2.

Textbox 2. **Inclusion and exclusion criteria applied in the scoping review.**

**Inclusion criteria**

- Target group: Clinical staff.
- Involvement: Participation in the development, design, piloting, and evaluation of artificial intelligence-based clinical decision support systems.
- Related approaches: Other related approaches, research and design strategies, or concepts, such as co-design, are often used interchangeably with participation and cocreation.
- Type of research: Primary research using different methods (eg, qualitative, quantitative, and mixed methods)
- Language of publications: English or German.

**Exclusion criteria**

- Participation: No evidence of a participatory research element.
- Target group: No relation to clinical staff.
- Thematic focus: Does not refer to AI-based clinical decision support systems.

### Study Selection

We used Zotero (a free and open-source literature management program) [34] as a bibliographic tool. The retrieved references were checked for duplicates and then transmitted to Covidence (software for managing and streamlining reviews, operated by Veritas Health Innovation Ltd) [35] for the screening steps. Moreover, 2 independent reviewers (TR and PG) screened all titles and abstracts separately for inclusion or exclusion. Disagreements were solved by including a third reviewer (CK or PK). The same procedure was then applied to full-text screening, which was carried out by 3 independent reviewers (TR, PG, and CH). Reasons for excluding a study were assessed in each of these steps.

### Data Charting Process

Two reviewers (TR and PG) collaboratively developed a data-charting form to determine which variables to extract. The form comprised seven sections: (1) metadata, (2) description of the contribution, (3) conceptual meaning and attribution, (4) theory and methodology, (5) implementation of participatory or cocreative processes, (6) evaluation and outcomes, and (7) miscellaneous. The data were independently charted by 3 reviewers (TR, PG, and CH), who then discussed the results and continually updated the data charting form in an iterative process. To ensure methodological consistency and quality, all reviewers screened 1 initial publication, discussed discrepancies, and jointly revised the data extraction manual before commencing formal screening. Disagreements were resolved by consensus or, when necessary, through consultation with the remaining authors (PK and CK), until full agreement was reached.

If information on specific participatory procedures, process characteristics, or content addressed within participatory activities was not explicitly described in a publication, this was documented as not reported (N/A) rather than coded as absent. This decision reflects the fact that publications often present only a selective account of the broader development process, and the absence of reporting does not necessarily indicate that a given activity did not take place or that a specific issue was not discussed.

In accordance with JBI guidance, the extraction form was iteratively refined compared with the version published in the review protocol [21,28,29]. Adjustments included expanding metadata categories, providing more detailed documentation of participants, and placing greater emphasis on participatory processes and contextual conditions. Items such as “Generic term for the participatory/co-creative process” and “Degree of participation/Grading” were not retained, as they were either not reported in the studies or would have required substantial interpretation. The final version of the extraction form is available in Multimedia Appendix 2.

### Data Items

Data were extracted on publication characteristics (eg, first author, year, journal, and country), study design (eg, clinical setting, target populations, and project phases), as well as key features of stakeholder engagement (eg, involved roles, recruitment procedures, and participatory or cocreative methods applied). Contextual and technical aspects were documented through detailed descriptions of the nature, function, and intended use of the AI-based CDSS. Barriers and facilitators to engagement were identified based on reported structural conditions and study design characteristics. Where applicable, we also extracted reported outcomes and perceived effects of participatory approaches on system development.

The extracted data were synthesized using a descriptive qualitative approach and organized deductively according to the 4 review questions, enabling a structured comparison of findings across studies.

## Results

### Overview

The systematic search yielded 4421 records from 4 scientific databases, supplemented by 40 additional records identified through citation searching. After removing 143 duplicates, 4318 records were retained for title and abstract screening. Of these, 64 studies were reviewed in full text. Twenty-seven were excluded from the study, primarily due to the lack of participatory elements (n=10) or the absence of an AI component (n=7). A total of 37 studies [36-72] satisfied the inclusion criteria and were thus incorporated into this review (Figure 1).

The 37 included studies represent a diverse range of research projects aimed at developing, testing, or evaluating AI-based CDSS involving clinical staff. Multimedia Appendix 3 provides an overview of key characteristics.

**Figure 1.**
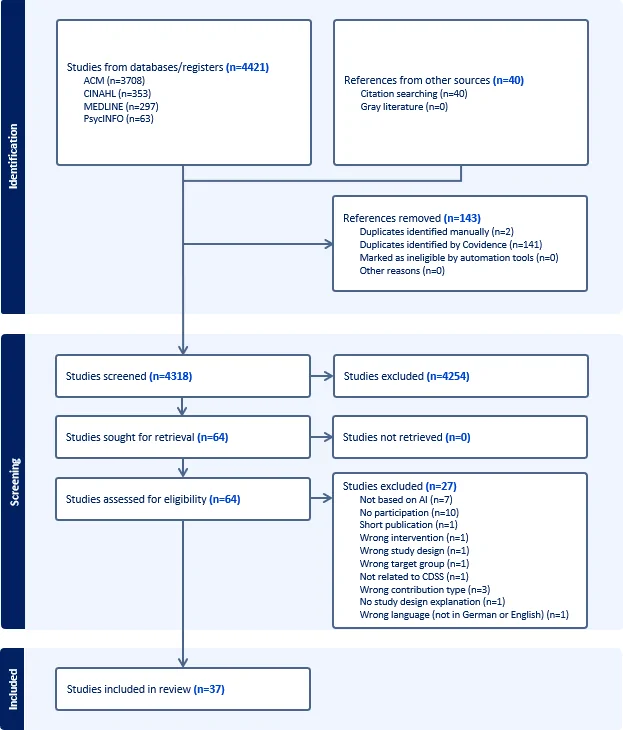
PRISMA (Preferred Reporting Items for Systematic Reviews and Meta-Analyses) flow diagram*.*

### Conceptions of Participation, AI, and CDSS

The analysis of the studies reveals considerable conceptual heterogeneity regarding key terms such as participation, AI, and CDSS.

The studies are sometimes not explicitly labeled as “participatory” or “cocreative” [36-47], yet they still report forms of stakeholder involvement in system development. More frequently, they adopt a user-centered design or “iterative or user-engaged” framing [38,39,41,48-59]. Explicit co-design, cocreation, or participatory-design terminology appears in 8 studies [60-67], whereas normative involvement labels such as “participation,” “consultation,” “collaborative partnership,” and “shared leadership” appear in 4 studies [68-71]. Across the included studies, however, explicit reference to participation remained limited, and descriptions of participatory processes were often brief or selective. In many cases, stakeholder involvement was identifiable through reported activities rather than through explicit conceptual framing or detailed methodological reflection. Accordingly, the labels used across studies did not consistently correspond to comparable forms of stakeholder involvement, and in several studies, interviews, focus groups, or usability procedures constituted the primary reported mode of involvement without being embedded in explicit co-design activities. Several included studies would not necessarily qualify as participatory in a strict definitional sense; rather, they reported more limited or consultative forms of stakeholder involvement. This ambiguity was closely related to the often sparse and selective reporting of involvement processes.

The systems described in the included studies primarily focused on predictive risk assessment in specific clinical contexts, such as stroke rehabilitation [60], heart failure [41], and wound healing [52]. Technological approaches ranged from machine learning [42,43,48,49,58-62,65,68-70] to deep learning [36,44,46,47,50,51,56,63,64,72] and hybrid models [37-39,55,57,66], while several studies did not specify in depth their underlying approach [41,45,53,54,67,71]. The level of technical detail varied considerably; while several studies provided comprehensive descriptions of model architecture and data sources [36,37,44,47,49,51,56,58,60,62,66,69], others remained vague or focused primarily on application aspects [38-40,42,43,45,46,48,52-55,57,59,61,63-65,67,68,70,71]. Similarly, the term “AI” was used inconsistently, ranging from precise model or explainable AI conceptualizations [36,37,39,44,47,50,51,53,54,56,58,60,62,63,65,66] to broad references to automated or learning-based systems [38,40,42,43,45,46,55,57,59,64,67,68,70,71].

Functionally, the systems were consistently described as tools to support clinical staff in making patient-specific decisions. Reported aims included improving decision quality and accuracy, early or more objective detection, prediction, or diagnostic support [40,42,44-47,49-52,55-60,64-66,68-71], facilitating integration into clinical workflows [38,41-48,51,53-57,59,61-66,68-71], and supporting shared decision-making between physicians and patients [36,43,58,59,67,72], as well as across multiple stakeholder groups, including physicians, patients, carers, and health professionals in integrated care settings [39,42].

### Diversity of Perspectives on the Underlying Clinical Problem

Several studies examined homogeneous groups of a single profession, such as psychiatrists [36,72], physicians [44,53,55,57,59], technicians [50], or nurses [52]. In contrast, other studies relied on heterogeneous, multiprofessional samples combining physicians, nurses, resident physicians, or therapists [37,39,41-43,45-49,54,56,58,61,63-67,69-71]. In most cases, the participating professionals mirrored the intended users of the system. Overall, the included studies predominantly involved physicians [36,38-41,43-49,51,53,55,57,61-66,68-72], followed by nurses [39-43,46-49,52,54,56,58,61,70,71]. Less frequently, therapists such as physiotherapists, occupational therapists, psychologists, or speech therapists were included [37,39,42,60,61]. In more detail, for example, Aquino [42] involved physiotherapists, occupational therapists, and speech therapists, Lee et al [60] involved occupational and physical therapists, Lee et al [37] mentioned therapists without specifying their discipline, Nemeth et al [61] involved physical, occupational, and respiratory therapists, and Timotijevic et al [39] included physical, occupational, speech therapists, and psychologists. Similarly, the participant groups also included specialized technicians [50] and, in another single instance, social workers and ventricular assist device coordinators [41], while several other studies further broadened the stakeholder spectrum by involving pharmacists, radiographers, managers, AI or HCI experts, patients, and patient or carer representatives [42,43,46,56,58,63-65,67,71].

Sample composition also varied by study phase. Needs assessments ranged from smaller expert groups or profession-specific samples, such as technicians, therapists, or physicians [50,51,60,69], to broader multiprofessional stakeholder constellations, for example, health and social care professionals, intensive care unit (ICU) and imaging staff, or oncology teams [42,45-47,64,66,70,71]. Physicians and nurses dominated design phases, sometimes also involving therapists [36-39,41,48,49,51,53,60-62,69], and, in some studies, being complemented by technical experts, patient and public contributors, or carers [43,56-59,63,65,67,71]. Evaluation phases ranged from profession-specific assessments to larger multiprofessional samples, involving physicians, nurses, residents, therapists, and other stakeholders [36,37,39-41,43-45,47,50-52,54-56,58-61,69,72]. In addition, some studies also involved nonclinical actors, such as designers, engineers, managers, AI or HCI experts, software developers, and nonclinical stakeholder groups including patient and public contributors, carers, and formal patient or carer representatives [43,51,56,63,65,67-69,71,72].

Across the studies, the breadth of stakeholder inclusion varied considerably. While several studies involved multiple professional groups relevant to the clinical workflow, others focused on only 1 professional perspective, even in contexts where broader interprofessional involvement appeared plausible. In most cases, participation remained closely aligned with the intended primary users of the CDSS. In contrast, actors indirectly affected by system use or embedded in adjacent care processes were less frequently included.

Multimedia Appendix 4 provides an overview of the participating groups, their roles, and the respective study phases. The column “participating groups” indicates the stakeholder groups involved in the studies, whereas “description of participants” outlines the actual study samples.

### Participation as a Process

The analysis of all included studies shows that user involvement in CDSS development is, in most cases, supported by theoretical, methodological, and empirical foundations. Many studies [36-38,40-42,47-52,54,55,58,60-62,66,68,71] referred broadly to concepts from human-computer interaction or general usability and user-centered design principles to motivate user involvement, typically in a general way and without linking these concepts to concrete participatory procedures. In total, 9 studies [39,56,59,62-65,67,70] explicitly connected theoretical models with the design of participatory processes. Methodological justifications were reported relatively consistently across the studies; however, they primarily referred to user-centered design activities, such as usability testing, think-aloud protocols, or requirements elicitation, rather than to participatory approaches in a stricter sense. In almost all studies [36-43,46-52,54-68,71,72], these methods were justified in functional terms, for example, to gather requirements, improve usability or explainability, or adapt the system to the clinical context. In contrast, explicit rationales for participatory involvement were rarely provided. However, eleven studies [47,51,56,58,59,61,62,64,66,67,71] included broader methodological reflections on the combination or sequencing of methods. Most included studies were empirically grounded, drawing on primary data or existing evidence to substantiate the value of user involvement.

The implementation of participatory and user-centered approaches varied across the studies. Several studies used interviews, focus groups, or workshops to engage clinical experts [36,38,39,41-47,49-51,53-61,63-72]. Additionally, some studies combined qualitative methods with usability and user experience (UX) techniques such as think-aloud protocols, task-based testing, and standardized questionnaires [37,44,45,47,48,50-53,55,58-60,62,65,66,69,72]. Iterative testing and feedback formats with repeated review cycles were also used [38-41,44,50,51,54,56-59,62,63,66,68,71]. Some studies also incorporated observational methods [40,41,50,51,60,61,72]. Finally, systems were evaluated in simulations or near-clinical scenarios [36,44,45,48,52,55,59,62,66,72]. Taken together, the reported activities ranged from formative inquiry (eg, interviews, focus groups, and observations) to more design-oriented collaborative formats and evaluation-oriented involvement (eg, think-aloud procedures, usability testing, and simulation-based feedback).

Reporting on participatory processes themselves was generally limited across the included publications, and details on power relations, possible biases, participation burdens, or supportive measures were often lacking. However, a few studies documented such aspects in more detail. Grant et al [70] reported introductory activities to support participants’ engagement and reflected on the practical burden of aligning participatory activities with clinical work schedules. Jung et al [54] likewise described supportive measures, including adapted session formats and separate walkthroughs for physicians and nurses to reduce hierarchical bias. Samimi et al [58] used structured walkthroughs to help participants engage with interface components step by step, while Fouad et al [63] deliberately balanced expertise across groups. In addition, Jung et al [53] reflected on how different participatory formats shaped participants’ ability to engage with future use scenarios. Overall, these remained exceptions, while most studies provided little information on how participation was enabled, how burdens were managed, or how power imbalances were addressed.

Several supportive conditions were identified. Recurrent facilitators included regular feedback loops or iterative refinement [37,39,41,47-51,54,56,59,62,68,71] and the use of real-world clinical cases [37,44,47,48,51,55,59,60], and interdisciplinary collaboration [38,46,49,51,58,59,63,66,68,69,71]. Some studies have also suggested that representations that are visually intuitive or cognitively accessible are beneficial [40,41,46,49,51,54,57,59,61,66,68]. Additional enabling conditions included the involvement of participants with domain-specific expertise [42,47,58,66], skilled facilitation and methodological triangulation [58,70], training, onboarding, and AI literacy support [53,54,56,70], close collaboration between clinical and technical or informatics teams [46,56,66,71], procedural flexibility to accommodate clinical workloads [46,53,54,71], and previous UX with related systems [47]. In addition, several studies reported compensation or protected time for participation, including paid working time or direct financial compensation [45,55,56,58,59].

At the same time, the studies highlighted several recurring challenges. Time and organizational constraints [36,38-41,48,51,53,54,60-62,65,66,69,70] frequently limited the depth of user involvement. Strategies to mitigate this included condensed feedback formats, asynchronous data collection or adjusted study designs to work around clinical schedules [36,38,41,45-47,53,54,56,61,69]. Skepticism and uncertainty toward AI, especially in early development phases, were also reported in the studies [39-42,44,45,47,51,59,62,62,67]. These concerns were addressed through explainability-oriented model or interface design, stakeholder-oriented design processes, and professional training or information adaptations [39-42,44,46,47,51,54,58,59,62,63,67]. Other methodological limitations or concerns included small or single-site samples, artificial test environments, recruitment bias, and prototype-stage evaluation [36,38,40,41,43-48,50,52-55,58-60,64,66,71,72]. These limitations were acknowledged in most cases, with some authors suggesting follow-up studies in real-world or multicenter settings [41,44-47,50,53,55,59,66,72]. In some studies, the inclusion of different disciplinary perspectives also led to coordination challenges and tensions arising from the diversity of stakeholder groups or the incomplete involvement of relevant groups [38,39,45-48,59,61,64,66,67,70,71]. Balancing model performance, explainability, and usability also emerged as a recurring trade-off [40,41,44-47,51,53,59,62,66,67].

Despite these challenges, many studies reported positive experiences with participatory development. User involvement supported the identification of practice-relevant requirements [36,38,41-49,51,53,57-59,61-67,70-72], facilitated contextual adaptation [39,41,42,44,46,47,50-52,55,58-61,63-66,69-71], and helped improve usability and interpretability through iterative testing and feedback [36,37,40,43,44,47,48,51,53-55,58,59,62,63,65,66,72]. In some studies, active cocreation appeared to help foster trust [37,39,40,44,51,56,58,63,65]. Additionally, several studies reported joint prioritization of features [38,39,47,49,51,53,57-59,63,66,67]. Several studies further described participatory processes as useful for surfacing mismatches between technical assumptions and clinical reasoning and for translating these into concrete design revisions, including shifts in system logic or interface structure [45-47,49,50,58,59,66,68].

Overall, the analysis highlights both the potential and the limitations of participatory development in the context of clinical AI. Although some studies offer practical strategies such as adaptive design processes or structured communication, persistent challenges—limited clinical validation, restricted generalizability, and structural resource constraints—show that important underlying tensions remain unresolved in the development of participatory AI systems in health care.

### Participation in the Technical Aspects of CDSS Design

The analysis shows that participatory methods can contribute in multiple ways to the development and refinement of AI components in CDSS. In 6 studies [37,46,50,56,64,69], the structured involvement of clinical professionals supported the identification of practice-relevant variables for model training. This was achieved through interviews, observations, journey mapping, contextual inquiry, workflow analysis, co-design workshops, focus groups, think-aloud procedures, iterative design processes, prototyping, and user studies. Additionally, participatory formats were used to co-develop visual and explanatory components that made AI outputs more transparent, interpretable, and clinically meaningful [37-39,41,44,48-55,58-60,62,63,65,66,72]. Methods used for this purpose included interviews [38,44,50,59,69], focus groups and iterative design sessions [41,49,57,63], think-aloud, observations and user studies [44,48,50,55,66], as well as simulation- or workshop-based formats (eg, using paper prototyping or probing cards [36,38,58,63,65], usability or cognitive walkthroughs [54,66], and heuristic evaluation [66]). Together, these approaches were used to elicit clinical reasoning and information needs, refine explanatory displays and interface elements, and test their comprehensibility and practical fit in more practice-oriented settings. Participatory methods also supported the alignment of AI-based components with clinical workflows and implementation contexts. Illustrative examples include simulation-based testing with standardized patients [36], which informed tool refinement and clinician training materials; focus groups and iterative design sessions [49], which shaped GUI adaptations; interviews and a workshop [38], which informed task models and user requirements; and iterative design activities across phases [41], which supported prototype refinement toward routine care.

A further contribution of participatory processes concerned data quality. Although data quality concerns were addressed in many studies, they were usually not framed as a distinct methodological focus of participation. In several studies [37,39,41-43,46-48,50,51,53,55,56,58,59,64,68,70,71], participants contributed to assessing the relevance, adequacy, and limitations of data used for AI development, for example, by identifying concerns related to clinically meaningful features, incomplete or unreliable routine data, erroneous inputs, limited dataset diversity, and problems of coding, documentation, or labeling. Methodologically, these data-related aspects were elicited through interviews and semistructured interviews [37,39,42,43,46,51,59,63,64], observations, journey mapping, contextual inquiry, and workflow analysis [46,50,64], individual evaluation sessions, user studies, and think-aloud-based testing [37,48,55], as well as iterative design, workshops, and fieldwork-based evaluation formats [41,50,56,58,59,64,70]. Additional contributions included therapist annotation [37], computational modeling and vignette-based assessments [39], and questionnaires and survey-based assessments [53,71].

Interpretability and explainability constituted a third area of participatory impact. In various studies [38,41,44,48-52,54,55,57-59,62,63,65,66,69,72], participants contributed to addressing these aspects within participatory processes. In several studies, explanatory components—such as visualizations, highlight features, or other explanation-oriented displays—were developed or revised with users through think-aloud–based evaluation and individual testing [44,48,55,66], focus groups and iterative design sessions [49,57,63], structured interviews and user observations combined with iterative design [50,59], and co-design sessions [58,62,65]. In the remaining studies [38,41-43,45,51-53,64,69,72], interpretability and explainability were addressed more indirectly by eliciting user requirements regarding transparency, data presentation, algorithmic reasoning, and the need for explanatory support. This was achieved through interviews [42,43,51,69], interviews and workshops [38,64], simulation-based testing and interviews [72], user feedback during prototype testing [52], iterative evaluation formats [41], and surveys or questionnaires combined with workshops or interviews [45,53].

In sum, participatory processes contributed not only to user-friendly interface design but also to the clinical relevance, data integrity, and transparency of AI components, provided that they are methodologically tailored to these aspects. Formats that engage clinical users not only retrospectively but proactively and iteratively in design and development decisions appear particularly effective.

### Participation for Ethical, Legal, and Social Implications

The studies included in this review address a variety of ethical, legal, and social implications (ELSI) emerging from the participatory development, testing, and evaluation of AI-based CDSS. While the term ELSI was rarely used explicitly, most studies included theoretical references and empirical insights into relevant aspects. In many cases, however, ELSI-related issues were only indirectly identifiable, as they were embedded in broader discussions rather than explicitly framed as ethical, legal, or social implications.

A recurring ethical principle is that AI systems should support, not replace, clinical decision-making—an aim explicitly stated in several studies [40,41,45,47,50,59,60,65,67,69]. The role of explainable AI is repeatedly emphasized, particularly in relation to transparency, trust, and the interpretability of automated recommendations [37,39,42-44,46,47,49,51,53,54,56,58,59,62,63,65-67,70]. Other common themes include adaptability to clinical workflows [40-42,46,59,60,68,69,71] and the potential of AI systems to enhance the visibility of underrepresented professional roles [40,42,46,56,60]. In addition, several studies highlighted the need for AI-based support to remain clinically meaningful and context-sensitive, for example, by adapting outputs to different users, tasks, and care settings rather than relying on overly generic recommendations [42,46,47,54,59,65]. Closely related to this, some studies also emphasized the importance of preserving clinician autonomy and avoiding overreliance on AI systems, so that AI was experienced as supportive rather than directive [36,45-48,50,61,66,67,72].

Clinicians voiced diverse expectations, concerns, and requirements. Trust in the technology emerged as a central theme; users demanded validated models, transparent recommendations, and adaptive visualizations, especially in situations of clinical uncertainty [39,40,42-44,47,49,51,53,54,56,58-60,62-67]. Preservation of professional autonomy was emphasized, with AI perceived as a source of reassurance or means of broadening perspectives rather than as an authoritative decision-maker [40,43-47,50,58,60,64,66,67,69]. Some studies reported that AI systems may help reduce subjective variability in clinical assessments [50,60]. Relatedly, participants in several studies described AI more concretely as a form of second opinion, an additional safety layer, or a supportive backup in situations of uncertainty, fatigue, or limited staffing [43,46,49,51,60,64]. Participants also stressed that useful AI support depends on contextual fit; outputs and interfaces needed to be clinically meaningful, interpretable, and adaptable to different users, tasks, and care settings, rather than generic or overly abstract [38,39,42,46,48-50,53,54,58-60,63-65,68,71]. Several studies further highlighted resistance to one-size-fits-all solutions and pointed to the need for role-specific interfaces or explanations tailored to different professional groups [47-50,54,58,61,65].

In most studies, fairness, bias, discrimination, and nondiscrimination were not explicitly discussed with users during participatory activities. Where these aspects were considered, they were mainly implicit, through design choices or technical adaptations. More direct participant-related considerations were evident in 4 studies [37,45,46,58] in which participants raised concerns about consistent, unbiased outputs; anchoring on AI recommendations and over-reliance; overly generic recommendations that may not fit diverse users and contexts; and uneven model performance across patient groups or clinical situations. Several other studies [36,38,40,51,52,60,61,72] showed more indirect consideration, for example, adapting functions to different experience levels [40,51], role-specific customization [38,60], integrating explainability features [52,60], or ensuring broad accessibility [36,61], or through accessibility-oriented design, safeguards against biased interpretation, dataset-related reflections, or broader ethical framing [42,47,56,59,63,67].

Questions of accountability and responsibility in CDSS-supported decision-making were seldom explicitly discussed with users. Where present, they were often addressed indirectly, for example, by emphasizing clinician autonomy or implementing design features to prevent over-reliance on AI [37,38,41,48,72]. More explicit reflections on role delineation, clinician responsibility, and the boundaries of AI support were evident in 9 studies [38,41-43,45,46,54,66,67], for example, through explicit reflections on role delineation, clinician responsibility, and the boundaries of AI support. Several studies [36,40,41,44,47,50,58-61,63] addressed the topic more implicitly, for example, by enabling users to review or reject AI suggestions [36,40], framing the CDSS as a supportive tool [36,47,58-60], or embedding mechanisms for critical review [41,44,61,63]. In Grant et al [70], related concerns focused mainly on clarifying responsibilities, routing documents, and tracking who submitted which data, rather than on accountability for AI-supported decisions in a narrower sense.

In total, 14 studies explicitly reported obtaining approval from a research ethics committee [36,38,46-49,55,56,59,61,64,69,71,72]. Funding sources were predominantly from public research grants [37-39,41,43-45,47-49,51-58,60,61,64-67,69-71]. Some studies additionally drew on philanthropic, foundation-based, or university-internal support [36,40,56,64,66,67,71,72], while Fouad et al [63] reported workshop-specific support from the Alan Turing Institute, although broader project funding was not specified. Moreover, 4 studies combined public funding with contributions from industry partners [36,50,62,72], whereas Staes et al [59] reported direct industry support without a clearly stated combination with public funding.

Of the total, 4 studies reported substantial conflicts of interest, as several authors were directly involved with the company developing the respective CDSS, either as shareholders, employees, directors, or scientific advisors, and some additionally received honoraria, sponsorship, or research funding from pharmaceutical companies [36,59,71,72]. In total, 13 studies explicitly declared that no conflicts of interest existed [38,39,43,45,48-50,54,56,57,64,69,70].

## Discussion

### Principal Results and Comparison With Previous Work

#### Conceptual and Semantic Heterogeneity

The analysis highlights the heterogeneity in how key concepts such as participation and AI are used. In most studies, participation was understood primarily in functional terms, for example, in relation to usability testing or user-centered design, and was rarely described in a theoretically grounded manner. One possible explanation for this functional framing is that most studies primarily focused on the technical development or evaluation of a CDSS. In this context, participation often served as a means to design systems for complex sociotechnical environments in a user-oriented way, rather than as an independent research goal or theoretically informed principle. Moreover, in many cases, participation was not a central topic of the publication and was therefore only briefly addressed or conceptually elaborated. However, previous work on co-design and participatory research emphasizes that participation should not be reduced to adapting technical systems to user needs. Still, it should also include the joint definition of goals and responsibilities [15,73].

The term “AI” was used inconsistently across the included studies and, in some cases, was only vaguely described. This inconsistency may also reflect the ambiguous nature of AI as a broad “suitcase” term, as well as the interdisciplinary character of participatory AI development, where technical, clinical, and social science perspectives intersect. Depending on the publication’s aim, the journal’s focus, and the target audience, the level of technical detail varies considerably. At the same time, the underlying computational architecture appears to play a rather minor role in shaping participatory processes. What seems to matter more is how system outputs are communicated, visualized, and integrated into clinical workflows [74].

Taken together, these observations point to a broader conceptual gap; participatory approaches in AI-CDSS are rarely linked to established theoretical frameworks such as value-sensitive design, participatory action research, or sociotechnical systems theory. Strengthening these foundations could help move participation beyond functional usability improvements toward a more epistemically and ethically informed design practice.

#### Perspectives of Professional Groups and Interprofessionality

The findings indicate that in most studies, the participating professional groups corresponded to the intended users of the respective system. The fact that physicians were involved far more frequently than nurses thus reflects the focus of many projects, which primarily aimed at supporting medical decision-making. At the same time, this emphasis points to persistent hierarchies in health care, where medical knowledge continues to be systematically valued above nursing and therapeutic expertise [75]. Previous reviews of CDSS also indicate that medical perspectives are primarily included in development processes, while nursing and therapeutic voices remain significantly underrepresented [76]. This not only limits the diversity of expertise but also overlooks the fact that professional groups differ markedly in their clinical responsibilities, information needs, and interaction with CDSS, differences that current designs often fail to address [47]. From a participatory perspective, this imbalance is problematic. Nursing professionals not only provide the majority of direct patient care, but are also largely responsible for continuous observation, documentation, and coordination [77]. Their decision-making reflects a complex process of observation, interpretation, and action that is deeply informed by experience, intuition, and contextual understanding, especially in complex care situations [47,77,78], underscoring the importance of incorporating such experiential expertise into system design. The subordinate role of nurses in the projects examined carries the risk that key routines and data sources essential to the functionality of CDSS will be insufficiently taken into account.

Beyond questions of representation, this imbalance also raises broader issues regarding whose knowledge is considered relevant in the development of clinical AI systems. Such dynamics have been described as forms of epistemic injustice, where certain forms of knowledge receive less recognition in processes of knowledge production [79,80].

In the context of AI-CDSS development, this means that selective involvement may also affect which knowledge enters technical design decisions. If nursing perspectives are less systematically involved, there is a risk that nursing knowledge is less strongly reflected in data selection, annotation, model assumptions, explanations, and visualizations.

This is particularly relevant because medical and nursing perspectives may differ in what they consider clinically meaningful information. Medical perspectives may foreground diagnosis-related parameters, treatment decisions, laboratory values, medication, or other structured clinical data, whereas nursing perspectives often include continuous observation, subtle behavioral changes, relational knowledge, everyday functioning, and contextual assessments developed through ongoing care. If such knowledge is not systematically included, AI-CDSS may reproduce a narrower representation of clinical reality, in which nursing observations, care-related documentation, and contextual information remain less visible. This risk is reinforced by existing digital infrastructures, as nursing knowledge is not always captured in a structured and reusable form in hospital information systems or electronic patient records. Previous work has shown that electronic documentation may limit nursing information exchange and that nurses’ clinical judgment may remain only partially visible in electronic records [81,82].

AI-CDSS that build on these data infrastructures may therefore inherit existing omissions in how nursing knowledge is digitally represented. This makes the systematic involvement of nursing perspectives in participatory AI-CDSS development particularly important, as it can help identify clinically relevant observations, documentation gaps, and care-related contextual information that may otherwise remain outside the system’s data and design logic. Without such involvement, selective knowledge inclusion may shape which variables are prioritized, how data are interpreted or annotated, which explanations are considered useful, and whether interfaces align with nursing workflows. As a result, AI-CDSS recommendations may be clinically or technically plausible, but still incomplete for concrete care situations if they do not reflect the observations and contextual knowledge of professionals who provide continuous bedside care. This may reduce the usefulness of the system for nursing users, limit its fit with interprofessional workflows, and make it less sensitive to early changes, care needs, or contextual risks observed in everyday nursing practice. At the same time, the reporting of participatory processes in the included studies was often too limited to assess systematically how decision-making power was distributed or whether such hierarchies were actively challenged during development. Further research is needed to better understand how participatory processes in AI development interact with existing knowledge hierarchies in clinical practice.

Multiprofessional approaches were used in some studies, but they remain the exception. Nevertheless, the small number of such approaches shows that interprofessional participation has not yet been systematically implemented. This means that projects run the risk of creating blank spots, for example, in relation to nursing routines, process-related care data, or interdisciplinary coordination.

On a positive note, some studies have also included nonclinical stakeholders such as software developers, data scientists, and experts in HCI. This illustrates that the development of CDSS is necessarily interdisciplinary and that technical and organizational knowledge must be combined with clinical practice. However, combining technical and medical expertise is insufficient if nursing perspectives remain marginalized. For sustainable participatory development, it is crucial that interprofessional and interdisciplinary collaboration is established and that nursing is represented equally [83,84].

#### Participation as a Process: Opportunities and Limitations

The studies examined used a wide range of methods to involve users, including interviews and focus groups, co-design workshops, usability tests, think-aloud protocols, and simulations. This diversity shows that various formats are available and can be selected based on project objectives, research questions, and the development phase. However, it is striking that participation was usually only documented in individual development phases, primarily in design or evaluation. In most cases, participatory efforts were mainly motivated by usability and acceptance-related goals rather than by ethical or epistemic considerations. Continuous involvement across multiple phases remained the exception. As a result, participation tends to be reduced to selective consultations rather than understood as an iterative, longer-term design process [13]. At the same time, the concrete forms of user involvement often followed a functional development logic rather than an explicitly participatory orientation. Against this background, it becomes necessary to critically question how “participatory” such involvement can be considered when it focuses on activities such as usability testing or data annotation. While these activities provide valuable input for interface design and data quality, they address relatively narrow aspects of system development. When involvement occurs mainly in these later stages, it focuses on components that are already specified; opportunities to influence the AI system’s overarching goals or conceptual assumptions are therefore limited. One possible explanation for these patterns is that, particularly when maintained over time, participatory involvement requires substantial time, personnel, and financial resources. Projects must therefore have the capacity to support such engagement, which often restricts their scope in practice [85].

At the same time, the absence of enabling activities or jointly conducted reflections on the participatory process suggests that important processual elements of participatory design were not taken up, even though such components are considered central for negotiating roles, responsibilities, and knowledge relations [31,86].

However, the included studies rarely described participatory processes in sufficient detail to assess how such dynamics were addressed in practice. Information on how participants were prepared for involvement, how possible burdens associated with participation were managed, or whether power asymmetries within groups were actively considered was largely absent. This also suggests that participatory involvement was often reported as inherently beneficial, while potential tensions or limitations of participation itself remained largely unexplored.

These observations also resonate with critical perspectives in participatory design research, which caution that participation can become instrumental or even extractive when stakeholders are primarily involved as sources of feedback rather than having meaningful influence on design decisions [18,19]. In such cases, participatory activities risk legitimizing predefined development trajectories rather than enabling shared decision-making about system goals and assumptions.

A related challenge concerns the limited and inconsistent reporting of participatory processes more broadly. This not only hampered comparability across studies but also made it difficult to trace how specific participatory activities contributed to particular design decisions or technical outputs. Although no reporting guideline currently exists specifically for participatory AI-CDSS development, recent guidance for the participatory development and evaluation of digital health interventions [87] may offer a useful orientation for improving transparency, comparability, and methodological learning in this field.

Beyond the design of individual system components, it would be valuable for participatory activities also to address the joint development of a CDSS’s goals, mechanisms of action, and contextual requirements [88]. Participatory methods such as participatory system mapping or theory of change-modeling offer suitable approaches for supporting these collective sense-making processes [89,90].

However, the studies also reported several concrete conditions that facilitated participatory processes in practice. Feedback loops, joint prioritization of features, working with real clinical cases, and interdisciplinary collaboration were described as particularly beneficial. These elements help to align systems more closely with clinical routines and improve usability. In addition, some studies provided protected time or financial compensation for participation. This should not be understood merely as a practical facilitation strategy, but also as a way of recognizing participants’ labor and practice-based expertise. Conversely, when participation depends on unpaid contributions in addition to routine clinical work, involvement may become more selective and more difficult to sustain over time [91,92]. At the same time, significant structural barriers emerged: time and organizational constraints, skepticism toward AI, small and poorly generalizable samples, and coordination problems within multiprofessional teams limited the depth and continuity of participation. These structural constraints should not be understood solely as logistical challenges, but as indicators of limited organizational capacity for innovation. In health care institutions, participatory activities usually add to existing clinical responsibilities; without protected time or dedicated resources, they compete with the immediate demands of routine care and are difficult to sustain [93]. Under such conditions, participation may become selectively accessible to professional groups with greater temporal autonomy, stronger institutional support, or more established links to research and development. Such findings are consistent with the literature, which indicates that (clinical) conditions often have a greater impact on participatory research than theoretical models [14,15].

These observations suggest that participatory development processes are closely shaped by their organizational and institutional contexts. While this review primarily focuses on participatory design processes, the findings also highlight the importance of implementation contexts and organizational conditions for the success of these approaches. Frequently reported barriers—such as time constraints, limited resources, and organizational misalignment—remain largely described at a surface level in the included studies. Interpreting these findings through the lens of implementation science may provide a more systematic understanding of why these challenges persist.

Established frameworks such as the Consolidated Framework for Implementation Research (CFIR) [94] and the NASSS (Nonadoption, Abandonment, Scale-up, Spread, and Sustainability) framework [95] highlight the role of factors, including organizational culture, workflow integration, stakeholder incentives, and system complexity, in shaping the adoption of digital health interventions. From this perspective, participatory design cannot be understood in isolation from the institutional environments in which it is embedded.

The limited attention to these dimensions in the reviewed studies suggests a gap in the literature. A similar pattern has been identified in recent research, which shows that real-world implementation of AI systems in health care remains rare and insufficiently documented [96].

Future research should more explicitly integrate participatory design approaches with implementation science frameworks to better understand how participatory practices can be sustained in real-world clinical settings. This includes examining how organizational structures, resource allocation, and policy environments facilitate or constrain meaningful participation. In practical terms, co-design workshops could be extended by an explicit reflection layer on implementation conditions, thereby linking participatory design work more closely to questions of adoption and sustained use. For example, a NASSS-informed implementation canvas could support stakeholders in identifying and reflecting on specific barriers and requirements for introducing digital technologies in clinical practice and in deriving possible solution approaches [97]. In addition, implementation-related reflection questions could be integrated into workshops and other participatory activities and guided by frameworks such as NASSS or CFIR. This would allow implementation considerations to be addressed throughout the participatory process rather than only after system development.

In summary, the findings indicate that participatory design in AI-CDSS remains fragmented and insufficiently theorized, with few studies explicitly connecting participatory methods to established frameworks such as value-sensitive design or sociotechnical systems theory.

#### Contributions to Technical Design

Participatory contributions focused primarily on peripheral technical levels, variable selection, data annotation, and explainability functions, while developers primarily determined the actual model and algorithm design. This pattern is by no means coincidental, but instead points to the unequal distribution of design power in the development process: clinical expertise is recognized as valuable for “enriching” data and testing interfaces, but not for the basic assumptions of modeling. The findings of Schwartz et al [76] support this observation: their review showed that clinical expertise was primarily involved in feature selection, annotation, and validation, while the core development of machine learning models was carried out almost exclusively by technical developers.

The unequal distribution of influence over design is also evident in how issues of explainability are handled. Although the involvement of clinical users in visualizations or case studies strengthened trust and comprehensibility, it was mostly reactive, focused on feedback on prototypes. Current work on explainable AI in the clinical context, on the other hand, emphasizes that explainability should not be treated as an afterthought but should be developed and negotiated with users as an integral design principle from the outset [98].

A related pattern becomes apparent in how data quality was managed. Data quality issues were only addressed in a participatory manner in a few projects. Where clinical experts were involved, annotations increased terminological precision, and user feedback verified the effectiveness of preprocessing steps (eg, feature selection), yet it is still striking that aspects of data quality were hardly addressed in many projects. This reveals a fundamental tension: while AI-based systems rely on the quality and relevance of their training data, this aspect is often ignored in participatory processes, even though clinical domain knowledge would be crucial for identifying and correcting systematic distortions and biases [85,99]. Such omissions point to deeper epistemic consequences. When data are treated primarily as a technical resource rather than as the outcome of social and clinical practices, their contextual meaning is lost [100-102]. In her analysis of atherosclerosis, Mol [103] demonstrated that the same disease is enacted differently across professional practices. Translated to the context of AI training data, this means that medical and nursing documentation do not merely offer different perspectives on the same “object,” but actively produce distinct versions of clinical reality. If annotations predominantly rely on medical perspectives, only a partial version of this reality is inscribed into the data. Nursing perceptions remain invisible, and this creates conditions under which forms of epistemic injustice may emerge.

Overall, this confirms a pattern observed in previous work: participation improves usability, data integrity, and trust, but remains limited to secondary technical levels [76].

#### ELSI

The findings suggest that ELSI aspects were addressed selectively across the studies. They can be further contextualized using established dimensions of trustworthy AI, including transparency and explainability, fairness and non-discrimination, accountability and governance, privacy and security, and human agency and oversight. Structuring the results along these dimensions highlights important asymmetries in how ethical, legal, and social implications are addressed in participatory design processes. Most attention was given to issues such as trust, transparency, and the preservation of professional autonomy. Trust itself was rarely addressed as an independent design objective, but rather emerged as a downstream consequence of transparency, explainability, perceived usefulness, and the preservation of professional autonomy. This emphasis is understandable, as it is directly linked to the practical usability of the systems. Without traceable outputs and the ability to retain clinical responsibility, CDSS are hardly compatible with everyday use. However, this focus narrows the ethical debate to trust and transparency, while other dimensions—such as fairness, bias mitigation, and accountability—receive far less attention. Yet these very aspects are particularly emphasized in international ethical debates on “trustworthy AI” [104]. Privacy and data protection considerations were occasionally mentioned but often remained implicit or underdeveloped. Similarly, aspects related to human agency and meaningful oversight were not systematically discussed. Čartolovni et al [105] show in their scoping review that security and transparency dominate, while fairness, accountability, and governance are considered critical but are often insufficiently addressed in practice.

This thematic narrowing is closely linked to the way ethical questions were negotiated within the development processes. ELSI aspects were rarely discussed with users in a participatory manner; instead, they were mostly addressed implicitly through technical design decisions or organizational frameworks. As a result, the role of clinical actors was often limited to accepting or evaluating transparency and trust functions, while broader ethical principles largely remained in the background. At the same time, the ethical relevance of participatory development depends on which perspectives become influential within these processes. If participation remains limited to certain groups, or if dominant voices prevail in discussions, there is a risk that existing assumptions will be reproduced rather than critically questioned [106].

This tendency becomes particularly evident in the case of responsibility, a central aspect of ethical decision-making. Several studies emphasized that CDSS should support decision-making rather than replace it. However, there was little participatory discussion on how responsibility can be distributed between humans and machines, and which structures can ensure this. This lack of engagement indicates that normative questions were rarely explicitly negotiated, instead being translated into technical or organizational solutions.

Taken together, these findings suggest that participatory AI-CDSS development has so far focused mainly on immediate usability, interpretability, and workflow integration, while broader ethical and governance dimensions remain insufficiently integrated into participatory design processes. As a result, there is a risk that “trustworthy AI” will be reduced to superficial characteristics, while deeper ethical tensions remain unaddressed. Making these dimensions more explicit within participatory processes may support a more comprehensive and transparent approach to AI development in health care.

### Strengths and Limitations

To our best knowledge, this is the first review to systematically examine approaches to involving clinical staff in the development, piloting, and evaluation of AI-based CDSS. By analyzing not only *when* involvement occurred across project phases but also *how* it was organized and *who* was engaged, the review provides a differentiated understanding of the forms of involvement. This highlights both the diversity of approaches and existing asymmetries in representation. The review thus offers a comprehensive overview and insights into how involvement can shape data, models, and normative principles, supporting the development of more inclusive and clinically meaningful AI systems.

This review has several limitations that should be considered when interpreting the findings.

A central limitation concerns the inconsistent and often selective reporting of stakeholder involvement, which hampered comparability and frequently obscured the extent, continuity, and influence of participatory processes. Understandings of involvement also varied across disciplines and contexts, underscoring the need for clearer methodological frameworks and standardized reporting practices. Another challenge concerned classification: because only a few studies explicitly described their approach using participatory terminology, inclusion decisions had to rely partly on reviewer interpretation, which carries a risk of subjectivity. Furthermore, most of the included publications were descriptive or conceptual, limiting the ability to draw quantitative inferences about the effectiveness of participatory approaches.

In addition, no formal critical appraisal of study quality was conducted. This approach is consistent with the exploratory purpose of scoping reviews, which aim to map the breadth and characteristics of a research field rather than assess the effectiveness of interventions. However, it implies that studies of varying methodological rigor were considered equally in the synthesis, which may influence the strength and interpretation of the conclusions.

A further limitation relates to the scope of the review. The search was restricted to English- and German-language publications from 2012 to 2026, which may have introduced language bias. Participatory design research has strong traditions in regions such as Scandinavia, and relevant studies published in other languages may therefore not have been captured. Moreover, the relatively small number of included studies compared to the initial search yield reflects the specificity of our review focus on AI-based CDSS with reported stakeholder involvement. While numerous studies address related areas such as digital health, health information systems, or co-design in adjacent technologies, participatory processes are often not explicitly described or are insufficiently reported. This underreporting itself represents an important finding of this review. At the same time, the focused scope of this review may have excluded relevant insights from adjacent domains, including participatory design in non-AI health technologies or broader co-design practices in health care. Future research may benefit from integrating these perspectives to develop a more comprehensive understanding of participatory approaches in the design of AI-based clinical systems.

Although this review covered the full spectrum from development to evaluation, none of the included studies provided systematic evidence on real-world implementation. The findings, therefore, mainly reflect early phases of development, piloting, and testing. The lack of implementation studies is likely related to the fact that AI-based CDSS have so far rarely been integrated into routine care [107], among other reasons due to restricted data access and regulatory hurdles [108], and broader socio-technical barriers that complicate implementation in health care settings [107]. Nonetheless, the review provides a comprehensive overview of existing practices and gaps across multiple disciplines.

### Conclusions and Recommendations

The development of AI-based CDSSs should integrate participatory processes continuously throughout all stages of system design and evaluation, rather than restricting them to isolated phases. Participation should address usability, data quality, bias, and ethical, legal, and social considerations. Co-design workshops, iterative prototyping, and shared evaluation dashboards can strengthen communication between developers and clinical users. Moreover, the reporting of participation and co-design remains insufficiently standardized, even though transparent documentation would be essential for contextualizing and critically reflecting on the research process. Although no reporting guideline currently exists specifically for participatory AI-CDSS development, existing guidance may offer useful orientation. This includes the *Best Practices in the Reporting of Participatory Action Research* [109] as well as the reporting guideline for the participatory development and evaluation of digital health interventions (ParDE-DHI) [87]. Such guidance underlines the need for studies to articulate more clearly how participation is conceptually understood, which theoretical assumptions underpin it, and how these assumptions are translated into concrete procedures. In addition, the use of technical reporting standards, such as TRIPOD+AI [110], can enhance transparency in model development and validation. Integrating participatory and technical reporting standards may help to make both user involvement and model transparency systematically visible, comparable, and reproducible. In addition, embedding deliberative and ethical reflection formats (eg, scenario discussions, data ethics cafés) can help translate high-level AI ethics principles into concrete design choices. It is equally important to involve diverse professional groups, particularly those with nursing and therapeutic expertise, to ensure that systems reflect the full spectrum of clinical routines and decision-making logics. As discussed above, participation has largely been framed through an HCI-oriented view of clinicians as “users,” yet their expertise would also be valuable for addressing implementation questions.

Future research should develop validated frameworks for participatory AI design that integrate ethical, organizational, and technical perspectives, thereby providing methodological coherence across studies and advancing the field. Longitudinal and comparative research could examine how different levels and forms of participation influence adoption, trust, and patient outcomes. In parallel, implementation studies in real clinical environments are needed to understand how to sustain participatory processes beyond project phases and embed them in institutional governance structures. Creating such frameworks and gathering empirical evidence will be essential to moving participatory design from a methodological aspiration to a practical standard for developing safe, responsible, and context-sensitive AI-CDSS.

## Supplementary material

10.2196/88368 Multimedia Appendix 1 Search Strategy.

10.2196/88368 Multimedia Appendix 2 Data extraction form.

10.2196/88368 Multimedia Appendix 3 Description of the studies.

10.2196/88368 Multimedia Appendix 4 Descriptive information on the participants in the studies.

10.2196/88368 Checklist 1 PRISMA-ScR checklist.

## Abbreviations

CDSS: clinical decision support systems
CFIR: Consolidated Framework for Implementation Research
ELSI: Ethical, legal, and social implications
HCI: human-computer interaction
ICU: intensive care unit
JBI: Joanna Briggs Institute
NASSS: Nonadoption, Abandonment, Scale-up, Spread, and Sustainability
ParDE-DHI: participatory development and evaluation of digital health interventions
PRISMA-ScR: Preferred Reporting Items for Systematic Reviews and Meta-Analyses extension for Scoping Reviews
UX: user experience
