# Supplementary material for: Participatory Design of AI-Based Clinical Decision Support Systems: Scoping Review
Source: JMIR Med Inform. 2026 Oct 5;14:e88368. doi: 10.2196/88368 (PMC13637979; doi:10.2196/88368)
Supplement: Supplementary file 1 [file medinform-v14-e88368-s001.pdf]

Multimedia Appendix 1 Search strategy

Search strategy

PCC	*#*	*Search string*	*Hits (Medline via PubMed, 03.03.23)*
Participation/participatory design/Co-Creation/Co-Design			
	1	participat* [all]	664,186
	2	co-creat*[all]	1,540
	3	cocreat*[all]	1,610
	4	co-design*[all]	1,706
	5	codesign*[all]	1,939
	6	co-research*[all]	408
	7	"patient involve*"[all]	3,159
	8	"public involve*" [all]	1,698
	9	"design research*"[all]	1,592
	10	"designs research*"[all]	21
	11	"research through design"[all]	11
	12	co-operat*[all]	12,813
	13	cooperat*[all]	262,736
	14	collaborat*[all]	259,752
	15	contribut*[all]	1,442,134
	16	user-led[all]	136
	17	"participatory design"[all]	572
	18	user-centered[all]	1,342
	19	user-centred[all]	458
	20	user-oriented [all]	231
	21	user-orientated [all]	12
	22	"community-based participat*" [all]	7,223
	23	"community-based research*"	1,388
	24	"human-centered design"[all]	436
	25	"human-centred design"[all]	94
	26	"Stakeholder Participation"[MeSH Terms]	3,865

	27	“Social Participation”[MeSH Terms]	3,362
	28	“Community Participation”[MeSH Terms]	47,551
	29	“Community-Based Participatory Research”[MeSH Terms]	5,911
	30	“User-centered Design”[MeSH Terms]	180
	31	“Social Inclusion”[MeSH Terms]	153
	32	OR/#1-#31	2,467,281
clinical staff			
	33	nurse*[all]	424,119
	34	“medical doctor*”[all]	5,551
	35	“medical staff”[all]	38,808
	36	“medical person*”[all]	6,905
	37	“health worker*”[all]	23,133
	38	“health staff”[all]	2,410
	39	“clinical staff”[all]	3,569
	40	“nursing staff”[all]	78,235
	41	“care staff”[all]	4,406
	42	“hospital staff”[all]	5,671
	43	nursing[all]	797,089
	44	therapist*[all]	42,117
	45	“practical nurse*”[all]	1,196
	46	“nurse practitioner*”[all]	26,190
	47	“care worker*”[all]	17,457
	48	physician*[all]	682,016
	49	“general practitioner*”[all]	56,069
	50	doctor*[all]	185,687
	51	“clinical practitioner*”[all]	1,043
	52	“health care provider*”[all]	37,059
	53	“Nursing Staff”[MeSH Terms]	69,641
	54	Nurses[MeSH Terms]	97,451
	55	“Medical Staff”[MeSH Terms]	28,786

	56	Personnel, Hospital[MeSH Terms]	95,043
	57	Physicians[MeSH Terms]	173,657
	58	*OR/#33-#57*	*1,852,022*
AI			
	59	“artificial intelligence*”[all]	51,367
	60	“artificial intelligence algorithm*”[all]	557
	61	“machine learning”[all]	60,369
	62	“machine learning system*”[all]	307
	63	“explainable artificial intelligence*” [all]	258
	64	“deep neural network*” [all]	4,869
	65	“deep learning”[all]	28,165
	66	“artificial neural network*” [all]	11,917
	67	“reinforcement learning”[all]	3,632
	68	“Human-Computer Interaction” [all]	1,330
	69	“Artificial Intelligence”[MeSH Terms]	166,652
	70	OR/#59-#69	201,559
CDSS			
	71	“clinical decision support system*”[all]	10,207
	72	“clinical decision support program”[all]	9
	73	“decision support system*”[all]	13,965
	74	“decision support tool*”[all]	3,270
	75	“clinical decision support”[all]	12,029
	76	“medical decision support” [all]	345
	77	“medical decision support system*” [all]	175
	78	Decision Support Systems, Clinical[MeSH Terms]	9,338
	79	“decision support techniques”[MeSH Terms]	81,985
	80	OR/#71-#79	97,702
	81	#32 AND # 58 AND # 70 AND #80	169
**+Filters**		#81 + Filters: English, German, Abstract, from 2012-2023	115
