# Supplementary material for: Participatory Design of AI-Based Clinical Decision Support Systems: Scoping Review
Source: JMIR Med Inform. 2026 Oct 5;14:e88368. doi: 10.2196/88368 (PMC13637979; doi:10.2196/88368)
Supplement: Supplementary file 2 [file medinform-v14-e88368-s002.pdf]

Multimedia Appendix 2 Data extraction form

Data extraction form

**Category**	**Sub-Category**
Metadata - Contribution	
	Author(s)
	Year of publication
	Country in which the study was conducted
	Title
	Journal/Source
	doi
	Study type / publication type
	Notes
Description of the contribution	
	contribution (topic; theme; indication; aims; purpose; objectives)
	Setting
	Target groups / population of the CDSS
	Participating roles/groups
	Total number of participants
	Description of participants
	Method of recruitment of participants
Conceptual meaning and Attribution	
	Attribution/Description of participation, co-creation and related terms/approaches (normative)
	Attribution/Description of the AI system type
	Attribution/Description of the clinical decision support system (CDSS)
Theory and Methodology	
	Theoretical, methodological and empirical foundations and justifications
Implementation of participatory/co-creative processes	
	Described participatory and co-creative methods
	Participatory study design
	Project phases
	Challenges/limitations, barriers
	Dealing with challenges/limitations, barriers
	Aspects of data quality
	Aspects of interpretability and explainability
	Considering different perspectives on the clinical problem when designing the CDSS
	Considered ethical, legal and sociocultural implications (ELSI)/recommendations for ELSI consideration
	Identified ELSI
	Aspects of fairness, bias and (non-)discrimination
	Accountability/responsibility aspects of clinical decision support systems
Evaluation and Outcomes	
	Supporting CDSS development through participatory methods
	Conditions for success, facilitating/supporting factors
	Outcomes and experiences
Miscellaneous	
	Miscellaneous

	Context and structural conditions
	Possible conflicts of interest for the author(s)
