# Supplementary material for: Participatory Design of AI-Based Clinical Decision Support Systems: Scoping Review
Source: JMIR Med Inform. 2026 Oct 5;14:e88368. doi: 10.2196/88368 (PMC13637979; doi:10.2196/88368)
Supplement: Supplementary file 3 [file medinform-v14-e88368-s003.pdf]

Multimedia Appendix 3 Descriptive information of each study included in the scoping review

Descriptive information of each study included in the scoping review

Study No.	First author (Year of publication)	Country (Data collection; Research team)	Journal / Conference	Study type	Main theme	Setting of the CDSS	Target group of the CDSS
1	Aquino (2025)	Data collection: UK; Research team: UK	Studies in Health Technology and Informatics (SHTI) – Global Healthcare Transformation in the Era of Artificial Intelligence and Informatics	Exploratory study, mixed-methods	The study investigates the essential requirements and feasibility of integrating a speech-based AI decision support tool into community-integrated care settings for the early and objective detection of Parkinson’s Disease progression.	community-integrated care, Parkinson’s care	clinicans; carers; people with Parkinson’s disease
2	Benrimoh et al. (2021)	Data collection: Canada; Research team: Canada, UK, USA	BJPsych Open	Evaluation study, mixed-methods	The study evaluates an AI-powered CDSS for major depression in a simulation center, focusing on its usability and impact on physician–patient interaction.	psychiatry; primary care	attending staff; residents
3	Blanes-Selva et al. (2022)	Data collection: Italy, Brazil, Spain, Greece, Scotland and Portugal; Research team: Spain, Brazil	Sage Journal: Digital Health	Evaluation study, mixed-methods	The study validates The Aleph PC, a machine learning-based CDSS for palliative care, by comparing its predictions to those of healthcare professionals and assessing usability, UX, and integration into clinical workflows through user-centered methods.	palliative care	general practitioners; hospitalists; palliative care; consultants; oncologists; neurologists; pulmonologists; nurses
4	d’Elia et al. (2025)	Data collection: UK; Research team: UK	BMJ Open	Evaluation study, qualitative	The study explores prescribing clinicians’ views on the usability, implementation, and shared decision-making relevance of a mock-up	Primary care	prescribing clinicians in primary care

					AI-based CDSS for prescribing in multiple long-term conditions.		
5	Fouad et al. (2024)	Data collection: UK; Research team: UK	ACM: BCS HCI '24: Proceedings of the 37th International BCS Human-Computer Interaction Conference/ACM	Design and development study, qualitative	The study further develops the XAI prototype CHERIE for chest radiology through a user-centred co-design process in order to identify user requirements for transparent, trustworthy, and clinically compatible interface design.	radiology	medical practitioners, specifically radiologists and clinicians involved in diagnostic decision-making
6	Grant et al. (2024)	Data collection: Germany; Research team: Germany	ACM: MuC '24: Proceedings of Mensch und Computer 2024	Needs analysis study, mixed methods	The study presents the results of an exploratory needs analysis using participatory methods to define practical use cases and assess staff expectations for developing an AI-supported system aimed at predicting aftercare needs and improving discharge management (DM) processes	discharge management; aftercare planning	hospital staff involved in discharge management / aftercare planning
7	Helman et al. (2021)	Data collection: USA; Research team: USA	International Journal of Medical Informatics	Design study, qualitative	The study explores clinician practices and perspectives to inform the early user-engaged design of a GUI prototype for predicting cardiorespiratory insufficiency and integrating it into clinical workflows.	predicting future cardiorespiratory insufficiency	nurses; nurse practitioners; physicians
8	Hussain et al. (2024)	Data collection: UK; Research team: UK	ACM: DIS '24: Proceedings of the 2024 ACM Designing Interactive	Design and Development study, qualitative	The study uses a co-design approach to develop and translate different human-AI interaction models into Wizard-of-Oz prototypes for clinical decision-making, in order to	obstetrics and diabetes care	clinicians in diabetes and obstetrics; in some interaction models also patients

			Systems Conference		explore how varying levels of clinician, AI, and patient involvement may shape shared decision-making.		
9	Hwang et al. (2022)	Data collection: Republic of Korea; Research team: Republic of Korea	Journal of Medical Internet Research	Evaluation study, qualitative	The study develops a user-centered CDSS to support polysomnographic technicians in reviewing AI-generated sleep staging results, focusing on clinically meaningful explanations and interface design informed by an extensive user study.	polysomnography	polysomnographic; technicians
10	Jin et al. (2020)	Data collection: China, USA; Research team: China, USA	ACM: Transactions on Computing for Healthcare	Evaluation study, mixed-methods	The study presents CarePre, an intelligent CDSS that uses deep learning to predict future diagnoses and treatment outcomes based on EHRs, emphasizing interpretability through interactive visualizations and evaluation with physicians through case studies and interviews.	decision making regarding diagnosis and treatment planning in clinical settings	senior physicians; pulmonologists; cardiologists; inexperienced; physicians; medical students
11	Jung et al. (2024)	Data collection: Germany, Spain, Poland, Belgium, USA, Italy; Research team: Germany	Studies in Health Technology and Informatics (SHTI) – Digital Health and Informatics Innovations for Sustainable Health Care Systems	Design study, mixed-methods	The study examines the early user-centered development of an explanation user interface for an AI-based clinical decision support system in intensive care, with a focus on eliciting and validating physicians' explanation needs across multiple centers and countries.	intensive care unit	ICU physicians
12	Jung et al. (2026)	Data collection: Germany; Research	JMIR Formative Research	Design and evaluation study,	This study aimed to evaluate the first iteration of the user-centered design and	intensive care unit	resident physicians; ICU nurses

		team: Germany		mixed-methods	formative evaluation of an explanation user interface (XUI) for an AI-based clinical decision support system intended to optimize mechanical ventilation in the intensive care unit, specifically identifying how differing information needs and priorities between physicians and nurses can be addressed through a layered design.		
13	Kaduwela et al. (2024)	Data collection: USA; Research team: USA	International Journal of Medical Informatics	Development study, mixed-methods	The study applies the Human-Centered Design for Embedded Machine Learning Solutions (HCDe-MLS) model to engage nurses in co-creating data labeling software (H2AI), which generates expert-labeled data to train a machine learning model for automated neonatal pain detection.	neonatal intensive care unit	NICU nurses
14	Köhler et al. (2025)	Data collection: Germany; Research team: Germany	JMIR Formative Research	Evaluation study, qualitative study	The study evaluates a high-fidelity prototype of an AI-based clinical decision support system for primary care, using user-centered usability testing with general practitioners to identify improvement needs and support adaptation to real-life clinical workflows in Germany.	Primary care	general practitioners
15	Lee et al. (2020)	Data collection: presumably USA, Portugal;	ACM: Proceedings of the ACM on Human-Computer Interaction	Design and Evaluation study, mixed-methods	The study co-designs, develops, and evaluates a machine learning-based decision support system for stroke	stroke rehabilitation	therapists (in stroke; rehabilitation; assessment)

		Research team:			rehabilitation assessment, collaborating with therapists to address their needs and enhance their decision-making in rehabilitation planning.		
16	Lee et al. (2021)	Data collection: USA; Research team:	ACM: CHI 21: Proceedings of the 2021 CHI Conference on Human Factors in Computing Systems	Development and Evaluation study, qualitative (with quantitative elements)	The study develops and evaluates an interactive AI-based system that collaborates with therapists for stroke rehabilitation assessment, integrating machine learning with rule-based models to enhance decision-making accuracy and feasibility in clinical practice.	rehabilitation for musculoskeletal and neurological diseases	therapists (in stroke; rehabilitation; assessment)
17	Leist et al. (2025)	Data collection: Germany; Research team: Germany	ACM: IUI '25: Proceedings of the 30th International Conference on Intelligent User Interfaces	Usability evaluation study, mixed-methods	The aim of the study is to evaluate an AI-driven CDSS for ophthalmologists in terms of efficiency, informedness, user experience, and perceived trustworthiness of its AI components.	ophthalmology	ophthalmologists
18	Mastrianni et al. (2025)	Data collection: USA; Research team: USA	ACM: Proceedings of the ACM on Human-Computer Interaction (CSCW)	Design and evaluation study, mixed-methods	The aim of the study is to design and evaluate an AI-based decision support system for time-critical medical events in the context of paediatric trauma care, and to investigate how two human-AI interaction strategies – information synthesis alone versus information synthesis with recommendations – affect decision-making regarding life-saving interventions.	pediatric emergency departmen	advanced practice providers; physicians making time-critical decisions during trauma resuscitation

19	Neff et al. (2024)	Data collection: Germany; Research team: Germany	Studies in Health Technology and Informatics (SHTI) – MEDINFO 2023 — The Future Is Accessible	Design study, qualitative	The study presents an initial user-centered design of an AI-based clinical decision support system for primary care, in which early interface prototypes are discussed with general practitioners in workshops to derive design recommendations for further development and routine use.	primary care	general practitioners
20	Nemeth et al. (2016)	Data collection: USA; Research team: USA	Journal of Cognitive Engineering and Decision Making	Design and evaluation study, qualitative	The study uses cognitive systems engineering (CSE) methods to analyze the cognitive work in a burn intensive care unit (BICU) and develop an IT system that enhances clinical decision-making, communication, and patient safety by addressing barriers to team coordination and efficiency.	burn intensive care unit (BICU); (tertiary care military academic medical center)	burn surgeons; medical intensivists; residents; bedside nurses; respiratory therapists; occupational therapists; nutritionists; wound care; infection control; anesthesiologists; etc.
21	Rafiq et al. (2024)	Data collection: Sweden; Research team: Sweden	International Journal of Medical Informatics	Exploration and design study, qualitative	The aim of the study is to explore the needs and preferences of physician managers regarding the role of predictive analytics in decision support for patients with the highly complex yet common combination of cardiovascular (heart) disease, kidney (nephrology) disease, and diabetes.	tertiary multidisciplinary ambulatory care	physician managers / healthcare managers involved in care planning and resource utilization for HND patients
22	Ren et al. (2020)	Data collection: The Netherlands; Research team: The Netherlands	ACM: CHI Conference on Human Factors Computing Systems: CHI EA '20: Extended	Design study, qualitativ	The study demonstrates how stakeholder engagement can inform the design of intelligent decision support tools (IDSTs) for occupational health	occupational health and safety; Human Total Care (HTC)	occupational health; physicians

			Abstracts of the 2020 CHI Conference on Human Factors in Computing Systems		practitioners, using the ConsultAI system as an example.		
23	Samimi et al. (2025)	Data collection: UK, USA, Canada; Research team: Belgium, Slovenia	ACM: CUI '25: Proceedings of the 7th ACM Conference on Conversational User Interfaces	Design and evaluation study, mixed-methods	The main contribution of this paper lies in the development and evaluation of an integrated clinical decision support system for diabetes risk assessment that combines interactive visualizations with conversation-based AI to improve healthcare professionals' understanding and confidence through scientifically grounded explanations.	diabetes care	healthcare professionals (who are involved in the assessment and management of Type 2 Diabetes)
24	Schütze et al. (2023)	Data collection: Germany; Research team: Germany	BMC Medical Informatics and Decision Making	Design study, qualitative	The study applies a user-centered design approach to develop an AI-based CDSS for German primary care, aiming to support diagnostic decision-making under uncertainty and ensure seamless integration into GPs' clinical workflows.	primary care; general practitioner practices	general practitioners
25	Shaeri et al. (2023)	Data collection: Iran; Research team: Iran	BMC Medical Informatics and Decision Making	Design, development and evaluation study, mixed-methods	The study presents the design, development, and evaluation of a smartphone-based CDSS to support early diagnosis and timely treatment of acute postoperative endophthalmitis, involving a multidisciplinary clinical team.	ophthalmology	physicians; patients

26	Sion et al. (2025)	Data collection: Unspecified; Research team: Estonia	ACM: ECCE '25: Proceedings of the 36th Annual Conference of the European Association of Cognitive Ergonomics	Exploratory design study, qualitativ	The study aims to use a user-centered co-design approach to explore clinicians' preferences for visual and textual AI explanations in early dementia risk prediction and to derive practical design implications for more transparent, trustworthy, and usable XAI interfaces in clinical contexts.	neurology	practicing clinicians, especially neurologists
27	Staes et al. (2024)	Data collection: USA; Research team: USA, Japan	Journal of the American Medical Informatics Association	Design study, qualitativ	The aim of the study is to use an iterative user-centered design process to develop a graphical interface for communicating ML-based 6-month survival prognoses for patients with advanced solid tumors to support oncologists' decision-making and serious illness conversations.	oncology outpatient clinic	oncologists
28	Tanguay-Sela et al. (2022)	Data collection: Canada; Research team: Canada, UK, USA	Psychiatry Research	Evaluation study, mixed-methods	The study evaluates a CDSS in a simulated clinical setting, focusing on physicians' perceived utility of the system in supporting treatment decisions and facilitating patient communication.	Major depressive disorder (MDD) treatment	primary care physicians (PCP); psychiatrists
29	Thieme et al. (2025)	Data collection: UK; Research team: UK, USA; India	ACM Transactions on Computer-Human Interaction	Exploration study, qualitativ	The study presents an in-depth case study to examine how a responsible, clinically useful AI application for chest X-ray–based nasogastric tube verification can be designed, evaluated, and integrated into clinical workflow from a human-centered perspective.	Intensive Care Unit (ICU) & Stroke Care (collaboration with Radiology)	ICU clinicians; reporting radiographers; radiologists

30	Timotijevic et al. (2020)	Data collection: Greece, Italy, Slovenia, UK; Research team: Greece, Italy, Slovenia, UK	BMC Medical Informatics and Decision Making	Exploration study, mixed-methods	The study applies a user-centered approach to develop a mobile CDSS prototype for Parkinson's disease, identifying clinicians' decision-making needs and evaluating the perceived value of patient-reported vs. mHealth-generated data.	Parkinson's care	neurologists; PD nurses; physiotherapist; patients; caregivers
31	Tookman et al. (2025)	Data collection: UK; Research team: UK	JMIR Cancer	Design study, mixed-methods	The study examines healthcare professionals' perspectives on challenges in retrieving ovarian cancer data from EHRs and describes the co-design of an integrated informatics platform to support clinical decision-making.	gynecological oncology	Multidisciplinary professionals working in gynecological oncology, including oncologists, nurses, radiologists, pathologists, and MDT coordinators.
32	vanBerkel et al. (2021)	Data collection: UK; Research team:	ACM Transactions on Computing for Healthcare, Vol. 2, No. 1	Design study, mixed-methods	The study investigates how visual markers in AI-assisted colonoscopy can support real-time polyp detection, focusing on clinicians' perceptions to inform the design of continuously adaptive visual decision support.	colonoscopies (gastroenterology)	endoscopists; gastroenterologists; colorectal nursing; personnel
33	Vogel et al. (2021)	Data collection: Germany; Research team: Germany	Public Health and Informatics - Proceedings of MIE 2021	Evaluation study, mixed-methods	The study develops and evaluates an AI-based clinical decision support system (CDSS) for chronic wound care, focusing on diagnostic and therapy support through iterative testing with nursing professionals.	chronic wound care	medical staff; nursing staff
34	Wang et al. (2019)	Data collection: Research team:;	ACM: CHI '19: Proceedings of the 2019 CHI	Methodological Study,	The study presents a user-centered XAI framework that aligns AI explanations with human	intensive care unit (ICU)	ICU clinicians

		Singapore and USA	Conference on Human Factors in Computing Systems	mixed-methods	reasoning to reduce bias and support decision-making in high-stakes settings like healthcare.		
35	Wu et al. (2025)	Data collection: USA; Research team: USA	ACM: CHI '25: Proceedings of the 2025 CHI Conference on Human Factors in Computing Systems	Design and evaluation study, mixed-methods	The paper presents the co-design and early evaluation of CardioAI, a multimodal AI-based system for symptom monitoring and cardiotoxicity risk prediction, and derives design considerations for clinically usable AI support in this context.	cardio-oncology	cardiologists; oncologists
36	Yang et al. (2019)	Data collection: USA; Research team:	ACM: CHI ’19: Proceedings of the 2019 CHI Conference on; Human Factors in Computing Systems	Design and evaluation study, qualitative	The study explores how to seamlessly embed a clinical Decision Support Tool (DST) into workflows to ensure acceptance and influence decision-making only when necessary.	VAD implant hospitals	Senior physicians; medical students; residents; other mid-level; clinicians
37	Zhang et al. (2024)	Data collection: USA; Research team: USA, China	ACM: CHI '24: Proceedings of the 2024 CHI Conference on Human Factors in Computing Systems	Evaluation study, qualitative	The aim of the study is to explore design requirements for AI systems that better support clinicians in the early diagnosis of sepsis and to develop and preliminarily evaluate a new human-AI collaboration paradigm for this purpose.		Physicians; nurses
