# Supplementary material for: Participatory Design of AI-Based Clinical Decision Support Systems: Scoping Review
Source: JMIR Med Inform. 2026 Oct 5;14:e88368. doi: 10.2196/88368 (PMC13637979; doi:10.2196/88368)
Supplement: Supplementary file 4 [file medinform-v14-e88368-s004.pdf]

Multimedia Appendix 4 Descriptive information on the participants in the studies

Descriptive information on the participants in the studies

Study No.	Involved stakeholder groups	Description of participants	Method of recruitment	Methods for participation
1	health and social care professionals	Needs assessment: 31 health and social care professionals	Purposive Sampling	Survey, focus group, interviews
2	psychiatrists; computer scientists; clinicians; patients; people with research skills in fields such as anthropology; psychiatry and family medicine attending staff; psychiatry and family medicine residents	Design assessment: 6 psychiatrists; Evaluation process: 11 psychiatrists; 6 family medicine residents; 9 family medicine attending staff members	Convenience Sampling	Simulations/scenarios (questionnaires, interviews, observations, feedback)
3	physicians; nurses	Design process: 7 general practitioners; 5 hospitalists; 1 palliative care consultant; 1 oncologist; 1 neurologist; 6 nurses	Snowball Sampling	Near-live simulation, questionnaire, think-aloud method with vignettes, observations, questionnaires
4	general practitioners/resident physicians; nurse prescribers; prescribing pharmacists; Patient Advisery Group (PAG) - either living with MLTCs (for or more long-term conditions), or caring for people with MLTCs	Evaluation process: 2 resident physicians; 3 nurse prescribers; 2 prescribing pharmacists; 9 people who either suffered from several chronic conditions themselves (four or more) or cared for people with such conditions, and who had experience of both common and rare conditions, as well as physical and mental health conditions	Purposive Sampling; Convenience Sampling	Interviews using a case vignette and a mock-up of the AI-based CDSS
5	healthcare professionals; AI experts; human-computer scientists; stakeholders from AI science, medical image analysis, clinical medicine, radiology, human-computer interaction, and social sciences	Recuirement analysis: healthcare professionals; AI experts; HCI researchers; Prototype validation and redesign: 30 multidisciplinary stakeholders; around one third with a relevant healthcare profession, the remainder researchers and scientists from AI, engineering/computer science, and HCI	N/A	
6	Discharge management staff; Physicians and nursing staff	Needs assessment process: 14 Discharge management staff; 3 physicians; 2 nursing staff	N/A	Participatory design workshops, brief questionnaires, expert interviews.
7	licensed clinicians; registered nurses; clinicians with prescribing authority (nurse practitioners); physicians with	Design process: 14 nurses; 4 nurse practitioners; 5 physicians	Purposeful Sampling; Snowball Sampling	focus groups

	experience in caring for hospitalized patients at risk for CRI			
8	Clinicians; HCI experts; AI safety experts ethics/law/psychology experts; patient representatives	Design and development process: Phase 1; 11 project team members (clinical experts, HCI experts, AI safety/ethics experts, etc.); Phase 2; 10 external clinicians (6 for Diabetes, 4 for Obstetrics); 5 patient panel members 5 project team members (3 clinical experts, 2 HCI experts); Phase 3; 4 external clinicians; 5 project team members (3 clinical experts, 2 HCI experts)	N/A	Focus group workshops, role-play simulations (with patient involvement panels), and "Wizard of Oz" prototyping, think aloud sessions
9	polysomnographic technicians with expertise in sleep staging.	Needs assessment: 10 technicians; Design process: 1 technician (1/10); Evaluation process: 9 technicians (9/10)	Convenience Sampling	Interviews, observations, iterative design process, think-aloud
10	senior physicians serving as associate directors; cardiologists; pulmonologists; physicians; professional designer	Needs assessment: 2 senior physicians serving as associate directors; Design process: 1 professional designer; Evaluation process: 3 senior cardiologists; 5 pulmonologists; 1 physician	N/A	Interviews, observations, iterative design process, think-aloud
11	physicians	Needs assessment: 4 physicians (1 senior,3 assistant); Design process: 9 physicians	N/A	Context analysis, user requirements prioritization workshops (using storyboards/mockups), online questionnaire
12	resident physicians; ICU nurses	Evaluation process: 4 resident physicians (anesthesiology); 4 ICU nurses	Convenience Sampling	Group usability walkthroughs, silent reflection, guided group discussion, individual quantitative assessments
13	NICU nurses; nurse scientists; human-centered design specialists; architects; data scientists; product manager	Requirements analysis: 6 NICU nurses; 2 nurse scientists; Design/development process: NICU nurses; nurse scientists; human-centered design specialists; architects; data scientists; product manager; Data labeling and model evaluation: 6 ICU nurses	N/A	Small video-call focus groups, co-creation / brainstorming sessions, mind mapping, prototyping, user testing and refinement, and data labeling workflows.
14	general practitioners	Evaluation process: 5 general practitioners	Purposeful Samping	Remote think-aloud tests with case-vignette tasks, post-session interviews, System Usability Scale questionnaire
15	therapists	Needs assessment: 9 therapists; Data collection: 15 post-stroke patients; 11 healthy patients; Design assessment: 2 therapists; Evaluation process: 7 therapists	Convenience Sampling	Interviews/focus groups, usability tests with questionnaires

16	therapists	Design process: 3 therapists; Dataset creation & annotation; 15 post-stroke patients; 11 healthy patients; 2 therapists; System evaluation: 7 therapists; Feature evaluation: 5 therapists	N/A	Interviews, user studies, usability tests with questionnaires
17	ophthalmologists	ophthalmologists	N/A	(Task-based) semi-structured interviews with think-aloud, questionnaires, usability assessment
18	fellows and residents from emergency medicine and surgery; surgeons; emergency medicine physicians; critical care physicians; practice provider	Design process: 2 surgery attendings; 24 emergency medicine attendings; 2 emergency medicine fellow; Evaluation process: 3 surgery attendings; 16 surgery residents; 1 nurse practitioner); 6 emergency medicine attendings; 4 emergency medicine fellows; 3 critical care attendings; 2 critical care fellows	Convenience Sampling; Snowball Sampling	Online survey, remote semi-structured interviews using mock-ups, within-subjects online experiment using simulated patient video vignettes, debriefing questions.
19	general practitioners	Design process: 5 general practitioners	N/A	Interviews, workshops
20	researchers; analysts; programmers; ML team members; nurses; medical technicians; residents; attending physicians; members of the client team	Design process and Evaluation process: 151 BICU clinicians; nurses (bedside, charge, wound care); residents; attending physicians; therapists (physical, occupational, respiratory)	N/A	Interviews, observations, workshops, usability evaluation based on scenarios
21	physician managers; physicians; healthcare managers.	Exploration process: 5 hospital managers; 4 HND physicians; Design process: 3 physician managers	N/A	Non-participant observations / shadowing, journey mapping, experience-based interviews, online co-design workshop.
22	product managers; software developers; data scientists; R&D consultants; physicians; occupational health physicians	Design process: 2 product managers; 1 software programmer; 2 data scientists; 1 research and development (R&D) consultant; 4 physicians; Design assessment: 2 product managers; 1 software programmer; 2 data scientists; 1 research and development (R&D) consultant; 4 physicians; Design evaluation process: 2 product managers; 1 software programmer; 2 data scientists; 1 research and development (R&D) consultant; 4 physicians	Convenience Sampling	Stakeholder walkthrough, co-design with group sessions, interviews
23	nurses; healthcare professionals (nurses, physicians, paramedics, diabetes educators)	Design process: 2 nurses; Evaluation process: 23 nurses; 2 physicians; 2 paramedics; 3 other healthcare roles.	Convenience Sampling	Online co-design sessions, structured walkthroughs, mixed-methods user study with scenario-based tasks, think-aloud, open-ended questions, and Likert-scale ratings
24	general practitioners	Design process: 5 general practitioners	Convenience Sampling	Interviews, workshop

25	retina fellows; ophthalmologists; medical information experts	Needs assessment: 6 ophthalmologists; 5 health information technology and medical informatics specialists; 5 fellow assistants and ophthalmology residents; Design process: 6 ophthalmologists; 5 health information technology and medical informatics specialists; 5 fellow assistants and ophthalmology residents; 2 application designers; Evaluation process: 7 ophthalmologists; 5 health information technology and medical informatics specialists; 5 fellow assistants and ophthalmology residents; 2 application designers	N/A	Interviews, UI review, usability questionnaire, focus groups
26	clinical researchers; data visualization engineer	Design process: 2 clinical researchers; 1 data visualization engineer.	Convenience Sampling	Card-based co-design sessions using probing explanation cards, think-aloud, physical annotation of the cards.
27	Oncologists; adult with red-green color blindness	Design process: 8 oncologists; 1 adult with red-green color blindness; Evaluation process: 5 oncologists	Purpose Sampling	Interdisciplinary team meetings, formative semi-structured interviews with case vignettes, think-aloud, contextual inquiry, heuristic evaluation, accessibility review, Likert-style rating questions
28	primary caregivers; psychiatry staff; psychiatry residents; physicians; research assistants; standardized patients (professional actors)	Evaluation process: 11 psychiatrists; 9 primary care physicians; 9 standardized patients (professional actors)	Convenience Sampling	Simulations/scenarios (questionnaires, observations), interviews
29	ICU staff (included junior physicians, registrars, consultants, and nurses); stroke care clinicians; imaging department staff (including consultant reporting radiographers and an A&E superintendent/reporting radiographer)	Exploration process: 8 physicians; 4 ICU nurses; 3 reporting radiographers.; 1 junior Clinical Fellow	Snowball Sampling; Convenience Sampling	On-ward observations / shadowing, semi-structured interviews.
30	consultant neurologists; hospital physicians; general practitioners; Parkinson's disease nurse specialists (PDNS); physiotherapists; occupational therapists;	Design process: 21 consultant neurologists; 3 Parkinson's disease nurse specialists; 9 general practitioners; 4 physiotherapists; 3 occupational therapists; 5 psychologists; 2 speech therapists; Design Assessment: 9 consultant neurologists; 1 consultant gerontologist; 2 general practitioners; Evaluation Process: 13 consultant neurologists; 2 general practitioners; 1 consultant gerontologist; 1 neurology resident; 1 researcher	N/A	Interviews, focus groups, vignette study using questionnaires, computer-assisted modeling of decision-making strategies

	psychologists; speech therapists			
31	Multiprofessional gynecological oncology professionals (including medical/clinical/gynecological oncologists, surgeons, nurses and clinical nurse specialists, radiologists, pathologists, allied health professionals, and MDT coordinators); data engineers/informatics experts; patient and carer reference group	Exploration process: 53 oncologists; 16 nurses; 10 radiologists; 13 other clinical roles; Design/development process: 3 medical oncologists; 1 gynecological oncology surgeon; 1 chemotherapy nurse specialist; 3 data engineers / informatics experts.	Convenience Sampling	National web-based cross-sectional survey, virtual co-design meetings, iterative clinician validation, patient and carer reference group meetings
32	endoscopists (Gastroenterologists, colorectal surgeons and endoscopic nurses); other clinical staff	Design process: 2 gastroenterologists; Dataset annotation; 1 domain expert (presumably a gastroenterologist; Evaluation process: 36 gastroenterologists	Purposeful Sampling; Convenience Sampling	Discussions, observations, and evaluation of the design by means of a survey
33	students with nursing experience	Evaluation process: 5 students with practical nursing experiences	N/A	Iterative test cycles (skills lab), simulation/freestyle scenarios (black box method)
34	ICU Clinicians; Human-Computer Interaction (HCI) Researchers; Cognitive Psychologists; Experts in Human Reasoning; Data Scientists; AI Specialists	Design process: 14 ICU clinicians	N/A	Think-aloud, scenarios, surveys for feedback
35	clinicians, including oncologists, cardiologists, and hematologists.	Design process: 11 clinicians; Evaluation process: 2 oncologists; 2 cardiologists	Convenience Sampling; Snowball Sampling	Individual remote participatory design sessions, cognitive walkthrough / heuristic evaluation, think-aloud, semi-structured interviews, standardized questionnaires (SUS, NASA-TLX)
36	biomedical researchers; cardiologists; surgeons; nurse practitioners; mid-level clinicians; clinicians from other medical domains	Design process: 1 cardiologist; 1 nurse practitioner; Design assessment: 9 cardiologists or surgeons; 8 mid-level clinicians; Generalizability assessment: 6 physicians	Snowball Sampling	Interviews, observations

37	clinicians	Design and evaluation process: 1 ICU staff nurse; 2 internal medicine physician; 2 emergency room physician; 1 ICU & emergency room physician	Snowball Sampling	Open-ended semi-structured interviews, heuristic testing with think-aloud, semi-structured post-study interviews.
